# Dual‐ vs. Single‐Antibiotic Loaded Cement for Hip Hemiarthroplasty: A Systematic Review and Meta‐Analysis

**DOI:** 10.1111/os.70056

**Published:** 2025-06-02

**Authors:** Cara Mohammed, Zuzanna Sandhu, Anjani Mahesh Kumar Cherukuri, Jeries Sayegh Adeeb Khouri, Kuruba Venkataramana, Aman Saswat Sahoo, Kabilesh Jothilingam, Seba Sayed Muhammed, Zain Elahi, Muhammad Ehsan, Lawrence Sena Tuglo, Raakesh Goalan

**Affiliations:** ^1^ Department of Orthopaedic Surgery Sangre Grande Hospital Sangre Grande Trinidad and Tobago; ^2^ Department of Neurosciences University Hospital Coventry & Warwickshire NHS Trust Coventry UK; ^3^ Department of Orthopedic Surgery Guntur Medical College Guntur India; ^4^ AlHussain New Salt Hospital As‐Salt Jordan; ^5^ Department of Orthopedic Surgery AIIMS Mangalagiri India; ^6^ School of Medicine and Dentistry, University of Central Lancashire Preston UK; ^7^ BAU International University Batumi Georgia; ^8^ Mid and South Essex NHS Foundation Trust Westcliff‐on‐Sea Essex UK; ^9^ Department of Medicine King Edward Medical University Lahore Pakistan; ^10^ Department of Nutrition and Dietetics School of Allied Health Sciences, University of Health and Allied Sciences Ho Ghana; ^11^ University of the West Indies St Augustine Trinidad and Tobago

**Keywords:** antibiotic‐loaded bone cement (ALBC), cemented arthroplasty, deep surgical site infections, surgical site infections (SSIs)

## Abstract

**Background:**

Antibiotic‐loaded cement (ALC) is often used to reduce the risk of surgical site infections (SSIs) in hip hemiarthroplasty; however, controversy exists regarding the use of dual antibiotic‐loaded cement (DALC) and single antibiotic‐loaded cement (SALC).

**Objective:**

This systematic review and meta‐analysis compare the efficacy of DALC and SALC for hip hemiarthroplasty.

**Methods:**

For this systematic review, a search was undertaken in the Cochrane Central Register of Controlled Trials, MEDLINE, Embase, and ClinicalTrials.gov. Grey literature such as ProQuest Dissertations and Theses Global (PQDT) was also explored. The inclusion criteria comprised randomized controlled trials (RCTs) or comparative observational studies, and patients undergoing hip hemiarthroplasty with DALC or SALC. Newcastle–Ottawa Scale (NOS) and RoB 2.0 tools were used for risk of bias assessment in observational and RCTs, respectively. Review Manager (RevMan, version 5.4.1; The Cochrane Collaboration, Copenhagen, Denmark) was used for statistical analysis. The primary outcome was the incidence of deep SSIs.

**Results:**

A total of five articles, including 28,418 participants, met the inclusion criteria. Three of the included studies were retrospective studies, one quasi‐randomized study, and one RCT. The primary outcome revealed that DALC was associated with a statistically significant reduction in deep SSIs compared to SALC (RR, 0.47; 95% CI, 0.29–0.76; *p* = 0.002; *I* ^2^ = 27%). Subgroup analysis based on the study design did not show a significant difference for deep SSIs (*p* = 0.29). The majority of the secondary outcomes, such as superficial SSIs, mortality, participants with ≥ 1 complication, or antibiotic use, did not show any significant difference. However, DALC significantly lowered the risk of any infection (RR, 0.55; 95% CI, 0.38–0.79; *p* = 0.001; *I* ^2^ = 27%).

**Conclusion:**

In conclusion, DALC can significantly reduce the risk of SSIs and the overall rate of any infection in hip hemiarthroplasty. A limitation of this study is that RCTs were pooled with observational studies, which decreased the power of analysis. Therefore, further research, including large RCTs, is needed to validate these findings.

## Graphical abstract

- DALC was compared to SALC in patients with hip hemiarthroplasty.
- A total of five studies involving 28,418 patients are included in the review.
- DALC significantly decreased the rate of deep SSI and any infection.
- No significant difference was found for other outcomes between the two groups.

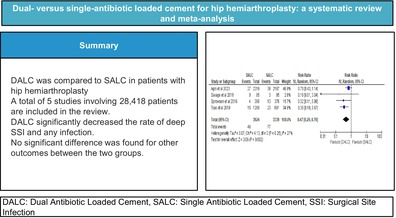

## 1 Introduction

Fractures around the hip in the elderly age group are associated with high morbidity and mortality. Hemiarthroplasty, a surgical procedure that typically replaces the femoral head and neck with a prosthesis, is commonly used to address complications from femoral neck fractures, allowing for early mobilization and preventing complications associated with prolonged immobility. In this procedure, the femoral component is fixed using bone cement made of polymethylmethacrylate, which functions as a “grout” despite its name [1]. Prosthetic joint infection (PJI) is a well‐recognized post‐operative complication that often requires multiple revision surgeries. This catastrophic complication predisposes to increased morbidity, extended hospital stays, and high healthcare costs [2]. Notably, the 1‐year mortality rate in patients with PJIs is significantly higher (43%–56%) compared to patients without PJIs, highlighting the critical need for effective preventive strategies [1, 2, 3]. A complex, multifactorial process involving the interaction of host, microbial, and environmental factors underlies the development of PJIs. The most common pathogens involved are *Staphylococcus aureus* and *Staphylococcus epidermidis* ; cases with gram‐negative organisms and polymicrobial infections are also seen [3].

A key factor in the progression of PJIs is forming a biofilm layer on the surface of prostheses, which leads to persistent and chronic infections. Within the biofilm, bacteria are metabolically inactive and cause recurrent infections, making their eradication particularly challenging [4]. Well‐recognized risk factors for PJIs include diminished immune response of the host, prolonged operative time, exogenous materials, and contamination during the surgery [5, 6]. The introduction of antibiotic‐loaded bone cement (ALBC) in hemiarthroplasty has had a substantial effect on the prevention of PJI, offering both prophylactic and therapeutic benefits against infections [7]. Introduced in the 1970s, ALBC delivers high local concentrations of antibiotics to a surgical site while minimizing systemic toxicity [8]. ALBC is effective in a wide array of orthopedic procedures, particularly in revision surgeries and the fixation of fractures where infection is suspected [9].

Gentamicin was the most commonly used antibiotic at the start of clinical practice, owing to its broad‐spectrum activity, stability when mixed with PMMA, and concentration‐dependent effects on bacteria [10]. This compound exhibits a post‐antibiotic effect; sustained antimicrobial activity persists even after drug levels have fallen below the minimum inhibitory concentration [11]. While this accounts for efficacy against bacterial growth, it is limited by the development of resistant strains and poor efficacy against some Gram‐positive bacteria [12]. These limitations are overcome by dual‐loaded bone cement utilizing antibiotics with synergistic mechanisms of action. Various studies have demonstrated the potential of incorporating antibiotics such as vancomycin, tobramycin, or clindamycin into bone cement to target specific pathogens or resistance patterns [13].

For instance, adding vancomycin to gentamicin broadens antimicrobial coverage to include methicillin‐resistant *S. aureus* (MRSA) and other resistant gram‐positive organisms [14, 15]. This synergistic action improves the bactericidal effect and minimizes the development of resistance [16]. This becomes particularly important in the context of PJIs, where polymicrobial infections by antibiotic‐resistant strains are common [17]. Comparative clinical trials have yielded variable results when evaluating single‐ vs. dual‐loaded antibiotic cements [18, 19]. While some RCTs have reported that dual‐loaded cement reduced the infection rate and improved efficacy in preventing PJIs, other studies failed to show an evident superiority for dual antibiotic‐loaded cement (DALC) [20, 21, 22]. While DALC offers certain benefits, there are safety concerns to consider. Increased antibiotic load can lead to systemic toxicity, especially if the elution of antibiotics is too fast or in a burst‐like manner. The mechanical properties of bone cement are predictably altered by the addition of powdered or fluid antibiotics to bone cement [23], negatively affecting the stability and longevity of the prosthesis [24].

Therefore, it is crucial to balance the antimicrobial effectiveness and mechanical integrity of the cement to achieve optimal results. Amidst the conflicting evidence from individual studies, a meta‐analysis is being conducted to compare dual and single antibiotic cements in hip hemiarthroplasty. This meta‐analysis aims to synthesize available evidence regarding the most effective and safe formulation for preventing PJI with ALBC in hemiarthroplasty, improving patient outcomes, and healthcare resource utilization. Understanding these findings could help optimize the formulation of ALBCs and individualize treatment strategies for patients.

## 2 Materials and Methods

This systematic review and meta‐analysis was conducted following the guidelines of the Cochrane Handbook for Systematic Reviews of Interventions and reported according to the Preferred Reporting Items for Systematic Reviews and Meta‐Analysis (PRISMA) [25, 26]. This study did not require ethical approval. The study protocol was registered in The International Prospective Register of Systematic Reviews (PROSPERO) under the identification number CRD42024569015.

### 2.1 Eligibility Criteria

The inclusion criteria were: (1) Study design: randomized controlled trials (RCTs) and comparative observational studies; (2) Patient population: Patients with hip fracture undergoing hemiarthroplasty; (3) Intervention: DALC; (4) Control: SALC as standard of care; and (5) Outcome: reporting at least one outcome of interest. The exclusion criteria were: (1) Studies conducted in vitro or on animals; (2) Studies comparing outcomes of DALC vs. SALC in total hip arthroplasty or arthroplasty of any other joints; (3) Drug elution studies; and (4) Studies with patients undergoing revision arthroplasty.

### 2.2 Information Sources

We conducted electronic searches of the following online resources from inception to July 2024 with no language or geographical restrictions: Cochrane Central Register of Controlled Trials (CENTRAL, via The Cochrane Library), MEDLINE (via PubMed), and Embase (via Ovid), ClinicalTrials.gov. We also explored grey literature sources such as ProQuest Dissertations and Theses Global (PQDT). The reference lists from the included articles and relevant systematic reviews were reviewed to find eligible studies. Forward citation tracking was employed using the Web of Science to look for further eligible studies citing any of the included articles and relevant systematic reviews. We employed a search strategy including a combination of keywords and Medical Subject Headings (MeSH) terms related to “antibiotic‐loaded cement,” “single antibiotic,” “dual antibiotic,” “infection,” “prosthesis,” and “arthroplasty.”

### 2.3 Study Selection

The results from the database search were imported into Rayyan [27]. After the duplicates were removed, each study was screened by at least two independent reviewers. The screening took place in two parts: title and abstract screening, followed by full‐text screening. Any disagreements between the reviewers were resolved through discussion.

### 2.4 Data Collection Process

The data from the included studies were independently extracted by two review authors into a structured Excel spreadsheet. The spreadsheet was piloted before the study to avoid discrepancies. Data items included study and patient characteristics (author name, year of publication, follow‐up period, number of patients, patient demographics such as age and sex, study arms, and comorbidities) and outcomes.

### 2.5 Outcome Measures

The primary outcome was the incidence of deep surgical site infection (SSI). The secondary outcomes were the incidence of superficial SSI, antibiotic use, mortality, and the incidence of at least one complication in the patient.

### 2.6 Risk of Bias Assessment

The Revised Cochrane Risk of Bias Tool for RCTs (RoB 2.0) was employed to assess the risk of bias in the RCTs among the included studies [28]. The Newcastle–Ottawa Scale (NOS) was used to assess the risk of bias in observational studies among the included studies [29]. The risk of bias assessment was performed by two review authors independently and rated as low, high, or some concerns for the RoB 2.0, and a star‐based rating system for NOS. Any disagreements between the reviewers were resolved by a third author.

### 2.7 Data Synthesis

Review Manager (RevMan, version 5.4.1; The Cochrane Collaboration, Copenhagen, Denmark) was employed for statistical analysis. The DerSimonian and Laird random‐effects model was used to perform meta‐analyses. Continuous outcomes were reported as mean difference (MD) with 95% confidence intervals. To ensure consistency in the analysis, we converted medians and interquartile ranges (IQRs) to means and standard deviations (SDs) using methods by Wan et al. [30]. Dichotomous outcomes were reported as relative risk (RR) with 95% confidence intervals (CIs). Heterogeneity was calculated for each synthesis by employing the chi‐square test and it is quantified by the *I* ^2^ statistic. The Cochrane Handbook for Systematic Reviews of Interventions was used to interpret *I* ^2^ values [25]. A *p* value of < 0.10 was considered significant.

Publication bias was planned to be estimated by constructing a funnel plot if there are at least 10 included studies in a meta‐analysis.

### 2.8 Subgroup Analysis

Subgroup analysis was conducted on the primary outcome based on trial design (RCTs and Observational). A *p* value of < 0.10 was considered significant for the subgroup differences [31].

## 3 Results

### 3.1 Study Selection

**FIGURE 1:**
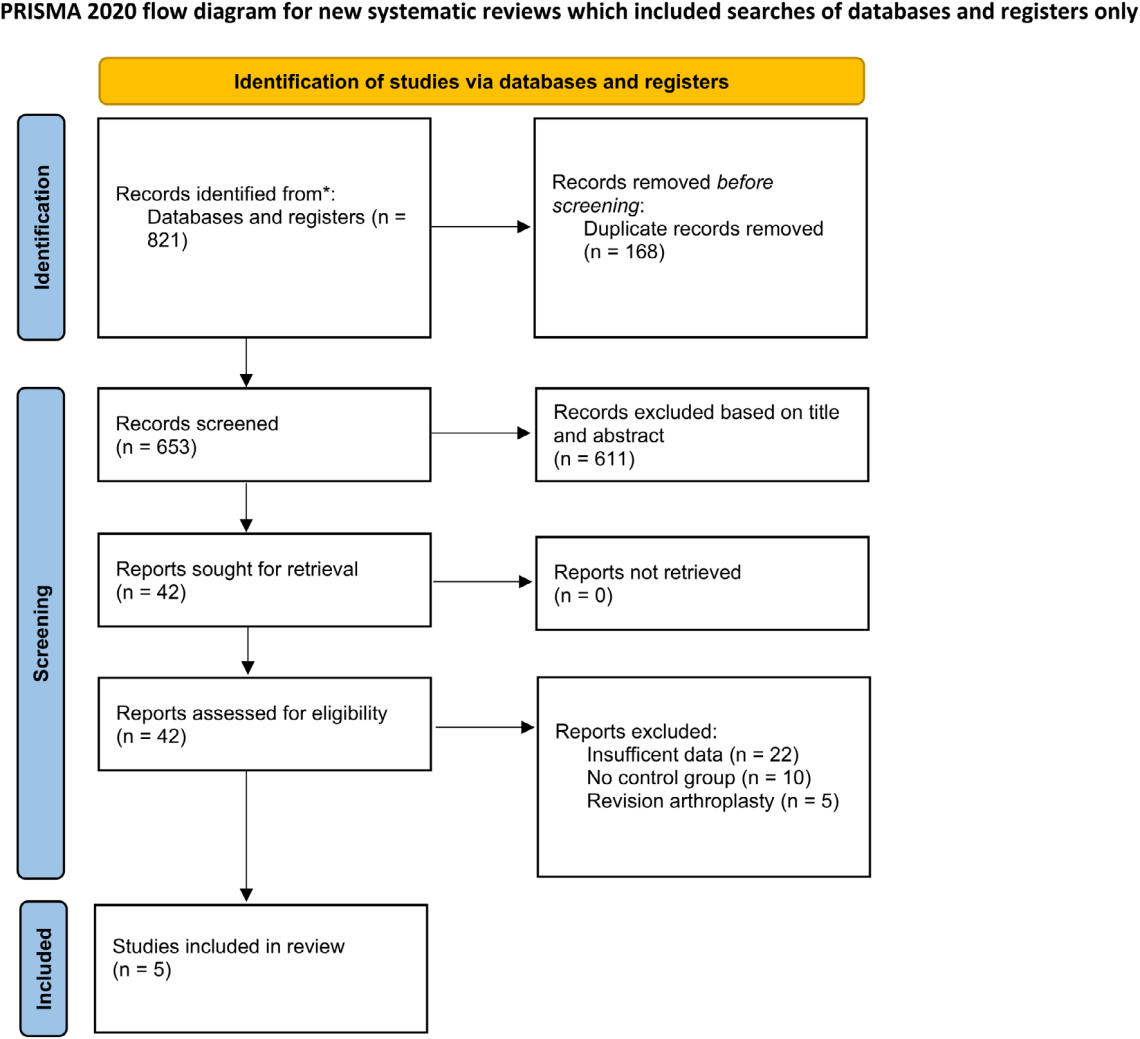
PRISMA flowchart.

The literature search yielded a total of 821 articles. Following deduplication, 611 studies were removed based on title and abstract. Forty‐two articles were included in the full‐length screening. Following a thorough assessment of full‐length articles, five articles were included in this systematic review and meta‐analysis. The study selection process is illustrated using a PRISMA flowchart (Figure 1).

### 3.2 Study Characteristics

A total of 28,418 participants were included in the studies, with the majority of participants in the control group (*n* = 22,743, 85.9%). The mean ages were above 80 years in both SALC and DALC groups. The proportion of male participants was 25%–33% in both groups. Three of the included studies were retrospective studies [18, 20, 32], one study was a quasi‐randomized study [19], and one was a randomized control trial (RCT) [22]. In the DALC group, three studies reported 1 g of clindamycin and 1 g of gentamicin as treatment medications, whereas two studies did not report drug type or dosage. Similarly, in the SALC group, three studies reported 0.5 g of gentamicin as the antibiotic used, whereas two studies did not report the type of drug or dosage. Follow‐up periods varied across the studies. Some studies reported no follow‐up period, while others had follow‐up durations ranging from 5 months to 5 years. Four studies were conducted in the United Kingdom (UK), whereas one study was from Germany. Table 1 summarizes the study characteristics of the included studies.

**TABLE 1:** Summary of the included studies.

Study authors and year	Study design	Country	Overall	Participants		Age (years)		Male participants		Dual antibiotic (intervention)	Single antibiotic (control)	Follow‐up
				Dual antibiotic (intervention)	Single antibiotic (control)	Dual antibiotic (intervention)	Single antibiotic (control)	Dual antibiotic (intervention)	Single antibiotic (control)			
Sprowson et al. 2016 [19]	Quasi‐randomized study	United Kingdom	848	400	448	82.96 ± 7.48	82.34 ± 7.69	101 (25.25%)	vs. 115 (25.67%)	1 g of clindamycin and 1 g of gentamicin	0.5 g of gentamicin	12 months
Savage et al. 2019 [32]	Retrospective cohort study	United Kingdom	206	98	108	84 (63–99)	83 (59–104)	29 (29.6%)	26 (24.1%)	1 g of clarithromycin and 1 g of gentamycin	0.5 g of gentamycin	12 months—
Agni et al. 2023 [22]	Randomized control trial	United Kingdom	4936	2483	2453	83.9 ± 7.4	83.8 ± 7.7	824 (33.2%)	821 (33.5%)	1 g of gentamicin and 1 g of clindamycin	0.5 g of gentamicin	5 months
Tyas et al. 2018 [18]	Retrospective study	United Kingdom	1941	—	—	—	—	—	—	—	—	No follow‐up
Szymski et al. 2023 [20]	Retrospective analysis of registry—cohort study	Germany	20,487	753	19,734	83.1 ± 7.77	84.2 ± 7.19	226 (30%)	5460 (27.7%)	—	—	5 years

### 3.3 Quality Assessment of the Included Studies

**FIGURE 2:**
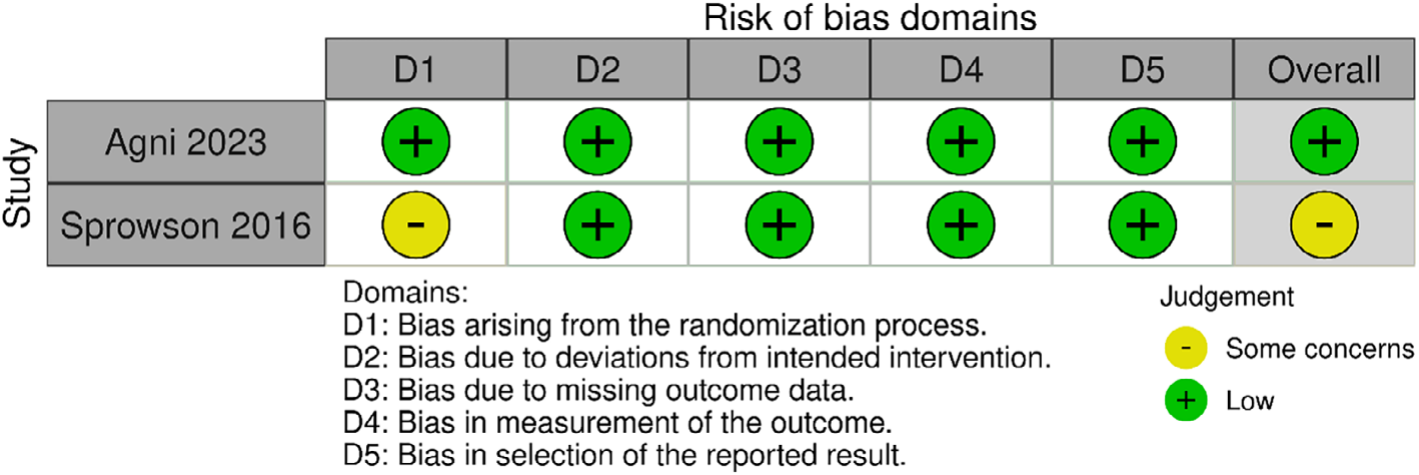
Summary of risk of bias assessment for each randomized control trial included in the meta‐analysis.

Risk of bias, measured by RoB 2.0 for RCT and the quasi‐randomized trial, showed an overall low risk of bias for RCT [22], whereas there were some concerns regarding the quasi‐randomized trial [19] due to bias arising from the randomization process (Figure 2).

The risk of bias, measured with NOS, revealed a quality score between 6 and 7 (Table S1). For two studies, the score showed a low risk of bias, whereas one study had an intermediate risk of bias [20]. The bias arose due to the lack of control for potential confounding factors in the included studies.

## 4 Results of the Meta‐Analysis

### 4.1 Primary Outcome: Deep SSI

**FIGURE 3:**
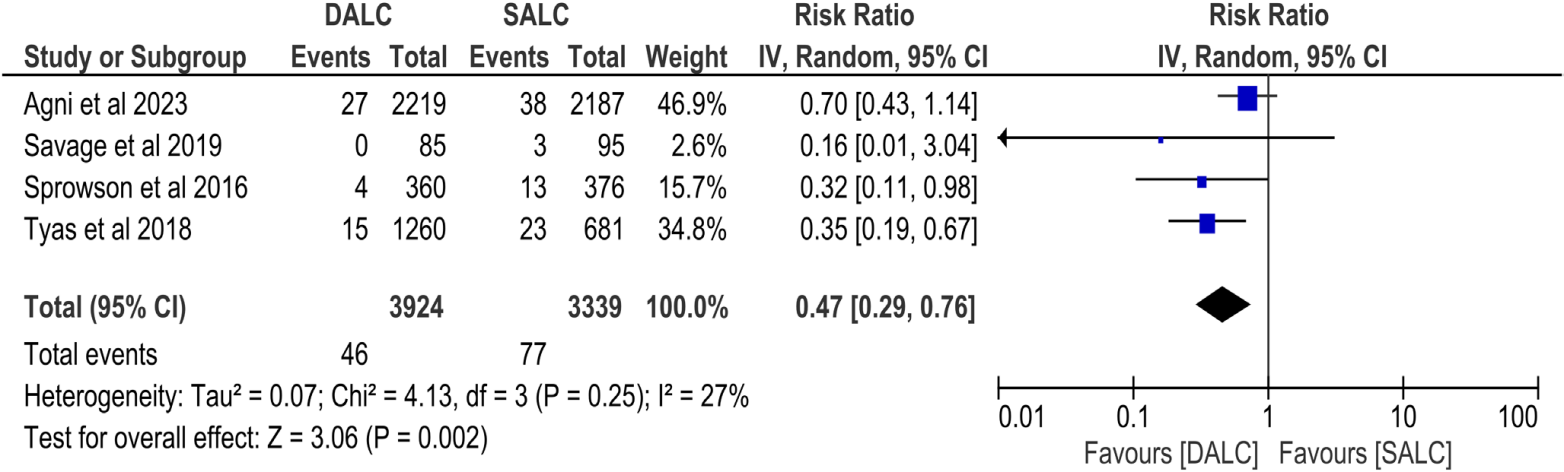
Forest plot of deep SSI.

Four studies reported deep SSIs and were included in the analysis. The DALC was associated with a statistically significant reduction in deep SSIs compared to SALC (RR, 0.47; 95% CI, 0.29–0.76; *p* = 0.002). The statistical heterogeneity between studies was moderate (*I* ^2^ = 27%) (Figure 3).

On subgroup analysis based on the study design, no significant difference was found between the two groups (*p* = 0.29; Figure S1).

### 4.2 Secondary Outcomes

#### 4.2.1 Superficial SSI

Only two studies reported superficial SSIs. There was no significant difference between the DALC and SALC groups regarding superficial SSIs (RR, 0.61; 95% CI, 0.17–2.23; *p* = 0.46). The *I* ^2^ value for the analysis was 36%, which indicates moderate heterogeneity (Figure S2).

#### 4.2.2 Antibiotic Use

There was no statistically significant difference regarding antibiotic use in the DALC and SALC groups (RR, 0.62; 95% CI, 0.33–1.15; *p* = 0.13). The interstudy heterogeneity was substantial (*I* ^2^ = 60%) (Figure S3).

#### 4.2.3 Mortality

Regarding mortality, no significant difference was observed between the DALC and SALC groups (RR, 0.96; 95% CI, 0.87–1.07; *p* = 0.49). The interstudy heterogeneity was low (*I* ^2^ = 0%) (Figure S4).

#### 4.2.4 Any Infection

The DALC group had a significantly low number of patients who experienced any infection (deep or superficial) compared to the SALC group (RR, 0.55; 95% CI, 0.38–0.79; *p* = 0.001). The statistical heterogeneity between studies was moderate (*I* ^2^ = 27%) (Figure S5).

#### 4.2.5 Participants With ≥ 1 Complication

There was no significant difference in DALC and SALC groups regarding participants with ≥ 1 complication (RR, 1.03; 95% CI, 0.97–1.10; *p* = 0.36). The statistical heterogeneity between studies was low (*I* ^2^ = 4%) (Figure S6).

## 5 Discussion

### 5.1 Summary of Main Findings

The findings of this systematic review and meta‐analysis, based on the analysis of 28,418 patients from five studies, showed that DALC is associated with a statistically significant reduction in deep SSIs and overall infection rates compared to SALC. The interstudy heterogeneity for both these outcomes was moderate. However, no significant difference was observed regarding superficial SSIs, antibiotic use, mortality, or the number of participants experiencing at least one or more complications. The subgroup analysis examining deep SSIs did not show any significant difference between randomized trials and observational studies.

This is the first systematic review and meta‐analysis conducted on DALC vs. SALC for hip hemiarthroplasty. Previously, a systematic review by Mohamed et al. assessed the efficacy of dual vs. single antibiotic cement; however, they did not perform a meta‐analysis [15]. Our findings are consistent with their results, which also demonstrated that dual antibiotics were associated with improved infection prevention. Their systematic review has several limitations. First, the majority of their included studies (70%) were in vitro studies, with limited clinical data available for deriving conclusions. Secondly, the included studies in their systematic review had low‐quality evidence, and only one level‐one study was included. Thirdly, they did not summarize overall findings from the included studies; rather, they presented findings and limitations from individual studies separately. The inclusion of a high‐quality RCT in the current systematic review and meta‐analysis is the main difference between our systematic review and meta‐analysis and the previous systematic review [15].

Our findings, however, did not align with the largest and only RCT published on the topic, which was also included in the present meta‐analysis [22]. The findings from 4936 participants included in the RCT showed no significant difference between DALC and SALC regarding any outcome measured. Although deep SSIs did not reach statistical significance in this RCT, the percentage of deep SSIs was lower in DALC compared to SALC (1.2% and 1.7%, respectively). However, our systematic review and meta‐analysis demonstrated a significant reduction in the DALC group, which can be attributed to the inclusion of observational studies in our analysis. Generally, small trials are prone to biases, and larger studies do not validate their findings. For example, two of the studies included in the meta‐analysis that demonstrated significant differences between DALC and SALC regarding deep SSIs had some risk of bias. Sprowson et al. [19] and Tyas et al. [18] had a moderate risk of bias due to problems with the randomization process and comparability for potential confounders, respectively.

In our systematic review and meta‐analysis, most of the studies used 0.5 g of gentamicin in the SALC group, while the DALC group employed a combination of 1 g of clindamycin and 1 g of gentamicin. The better outcomes observed in the DALC group for preventing deep SSIs and infections can be attributed to the synergistic effect of gentamicin and clindamycin. The gentamicin‐impregnated SALC has demonstrated positive effects against *Pseudomonas, Staphylococcus*, and *Enterobacter*. However, DALC with gentamicin and clindamycin provides additional cover to *Staphylococcus*, *Streptococcus*, and anaerobes [32]. Furthermore, evidence from in vitro studies has demonstrated that DALC with 1 g of gentamicin and 1 g of clindamycin inhibits the growth of bacterial colonies for 672 h compared to 48 h seen in SALC with 0.5 g of gentamicin [33].

## 6 Strengths and Limitations

This is the first systematic review and meta‐analysis to investigate outcomes for DALC vs. SALC in hip hemiarthroplasty. A comprehensive search using several registries and databases was conducted to identify RCTs and observational studies that met our inclusion criteria. The main strength of this systematic review and meta‐analysis is the inclusion of the first RCT to date in our analysis. However, there are several limitations to consider when interpreting the findings. We have included only five studies and pooled the RCT with observational studies, thus decreasing the power of analysis. Nonetheless, a subgroup analysis based on the type of studies was performed for the primary outcome. Two of the included studies did not specify the antibiotic doses in the SALC and DALC groups, making it impossible to assess the impact of different antibiotics and their dosages. We could not assess the potential relationship between antibiotic dosage and the mechanical properties of bone cement, including the risk of periprosthetic fracture. Another limitation is the variability in follow‐up duration; follow‐up of more than 1 year is recommended to identify long‐term outcomes of DALC and SALC in hip hemiarthroplasty. Additionally, except for one study published in Germany, all studies were from the UK, limiting the generalizability of the findings. Finally, we used aggregate‐level data in our analysis as individual patient data was not available.

## 7 Implications for Practice and Research

The findings of our systematic review and meta‐analysis, based on data from 28,418 patients, demonstrated that DALC can significantly reduce the deep SSIs and overall infection rate compared to SALC in hip hemiarthroplasty. Clinicians should consider the potential benefits of combining gentamicin and clindamycin in DALC for hip hemiarthroplasty, as suggested by the findings. The synergistic effect of these antibiotics may offer superior protection against deep SSIs compared to gentamicin alone in SALC.

Currently, there is a paucity of research comparing DALC with SALC in hip hemiarthroplasty, as only five studies have been published. Furthermore, only one RCT has been published so far. Therefore, there is a need for more high‐quality evidence from RCTs to investigate the effects of individual drugs and their dosage. This will allow for a more accurate assessment of the impact of specific antibiotic regimens on outcomes, facilitating more precise comparisons and clinical recommendations. Future studies should explore whether variations in antibiotic load impact cement integrity and long‐term prosthesis survival. There is also a need for individual patient data meta‐analysis on this topic. As the studies included in this meta‐analysis are either from the UK or Germany, future studies should be conducted in the USA and other parts of the world to ensure the generalizability of the findings. Given the variation in follow‐up durations observed in the included studies, future research should also standardize follow‐up periods to ensure consistency in outcome assessment.

## 8 Conclusion

DALC is shown to significantly reduce deep SSIs and overall infection rates in hip hemiarthroplasty. However, the beneficial effect of DALC was not demonstrated in other outcomes, including superficial SSI, antibiotic use, mortality, or the number of participants experiencing at least one or more complications. Further research, including large RCTs, is needed to validate the findings of this systematic review and meta‐analysis.

## Author Contributions

**Cara Mohammed:** conceptualization, data curation, formal analysis, investigation, methodology, software, writing – original draft. **Zuzanna Sandhu:** conceptualization, data curation, formal analysis, investigation, methodology, software, writing – original draft. **Anjani Mahesh Kumar Cherukuri:** investigation, methodology, writing – original draft, data curation. **Jeries Sayegh Adeeb Khouri:** formal analysis, investigation, methodology, writing – original draft. **Kuruba Venkataramana:** investigation, methodology, resources, writing – original draft. **Aman Saswat Sahoo:** investigation, validation, visualization, writing – review and editing. **Kabilesh Jothilingam:** data curation, formal analysis, investigation, methodology, writing – original draft. **Seba Sayed Muhammed:** data curation, writing – review and editing. **Zain Elahi:** project administration, resources, supervision, writing – review and editing. **Muhammad Ehsan:** conceptualization, data curation, formal analysis, investigation, methodology, project administration, resources, supervision, validation, visualization, writing – original draft, writing – review and editing. **Lawrence Sena Tuglo:** methodology, resources, supervision, writing – review and editing. **Raakesh Goalan:** investigation, resources, supervision, writing – review and editing.

## Ethics Statement

We confirm that we have read the Journal's position on issues involved in ethical publication and affirm that this report is consistent with those guidelines.

## Conflicts of Interest

The authors declare no conflicts of interest.

## Supporting information


**Data S1.** Supporting Information.

## Data Availability

Data will be provided on reasonable request from the corresponding author.
